# Folate and retinal vascular diseases

**DOI:** 10.1186/s12886-023-03149-z

**Published:** 2023-10-13

**Authors:** Jinyue Gu, Chunyan Lei, Meixia Zhang

**Affiliations:** 1 Department of Ophthalmology, West China Hospital, Sichuan University, 610041 Chengdu, China; 2 Research Laboratory of Macular Disease, West China Hospital, Sichuan University, 610041 Chengdu, China

**Keywords:** Folate, Homocysteine, Retinal vascular occlusions, Diabetic retinopathy, Age-related macular degeneration

## Abstract

Folate, a pteroylglutamic acid derivative, participates in fundamental cellular metabolism. Homocysteine, an amino acid, serves as an intermediate of the methionine cycle and can be converted back to methionine. Hyperhomocysteinemia is a recognized risk factor for atherosclerotic and cardiovascular diseases. In recent decades, elevated plasma homocysteine levels and low folate status have been observed in many patients with retinal vascular diseases, such as retinal vascular occlusions, diabetic retinopathy, and age-related degeneration. Homocysteine-induced toxicity toward vascular endothelial cells might participate in the formation of retinal vascular diseases. Folate is an important dietary determinant of homocysteine. Folate deficiency is the most common cause of hyperhomocysteinemia. Folate supplementation can eliminate excess homocysteine in plasma. In in vitro experiments, folic acid had a protective effect on vascular endothelial cells against high glucose. Many studies have explored the relationship between folate and various retinal vascular diseases. This review summarizes the most important findings that lead to the conclusion that folic acid supplementation might be a protective treatment in patients with retinal vascular diseases with high homocysteine or glucose status. More research is still needed to validate the effect of folate and its supplementation in retinal vascular diseases.

## Introduction

Folate, a collective term for a family of water-soluble vitamins (vitamin B9), plays an essential part in one-carbon metabolism, which primarily involves the metabolism of key amino acids and nucleic acids [1]. It has two forms, including naturally occurring folate and folic acid [2]. Natural folate was first found by Lucy Wills in 1931. It was extracted from yeast and could prevent and cure the macrocytic anemia that often occurred in late pregnancy [3]. Folic acid, an oxidized and stable synthetic form of vitamin B_9_, can only be obtained from fortified food, supplements, and pharmaceuticals. Mammals cannot self-synthesize folate. Hence, folate can only be obtained through dietary sources and folate supplementation. Folate deficiency is often caused by inadequate dietary intake, which can lead to several human disorders, such as megaloblastic anemia and neural tube defects in fetuses [4], as well as cardiovascular disease, certain types of cancer, and cognitive diseases [5, 6]. Folate, as a cofactor, plays an important part in one-carbon metabolisms which is required in multiple biological processes including DNA synthesis, DNA reparation and production of methionine by methylation of homocysteine.

Homocysteine, an amino acid, serves as an intermediate in the methionine cycle. It can be converted back to methionine or transsulfrated to cysteine. 5-Methyltetrahydrofolate (5-mTHF) serves as a cofactor for the remethylation of homocysteine into methionine via methionine synthase, a vitamin B12-dependent enzyme. Deficiency of folate or vitamin B12 can lead to the elevation of plasma homocysteine levels [5]. In the early 1990s, elevated plasma homocysteine levels became a recognized risk factor for atherosclerotic disease [7]. In addition, elevated plasma homocysteine levels have been reported as a risk factor for stroke, dementia, and retinal vascular occlusive diseases [8–10]. Moreover, homocysteine was found to induce endothelial dysfunction in hyperhomocysteinemia [11]. Many retinal vascular problems occur in hyperhomocysteinemia such as retinal vascular occlusions and diabetic retinopathy. Retinal vascular diseases are abnormalities that affect the blood vessels in the eyes and are the main causes of visual impairment and blindness worldwide [12]. Many studies have found that low plasma folate levels and elevated homocysteine levels are independent risk factors for some retinal vascular diseases, including central retinal vein occlusion (CRVO) and diabetic retinopathy (DR) [13, 14]. Moreover, daily folic acid supplementation could effectively reduce the concentration of homocysteine and may protect vascular endothelial cells [15]. In this review, we discuss the biochemistry of folate, its role as a risk factor for retinal vascular diseases, and treatment studies with folate supplementation.

## Biochemistry of folic acid

**Fig. 1:**
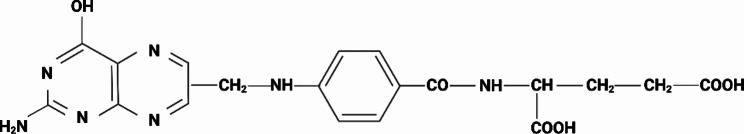
Chemical structure of folic acid

Folate is a collective name for a family of water-soluble vitamins (vitamin B9) that have similar properties and share a similar structure with pteroylglutamic acid [16]. It includes natural folate and folic acid. In nature, folate exists in leafy green vegetables, fresh fruits, beans, nuts, liver, and kidneys, mainly as 5-methyltetrahydrofolate and formyltetrahydrofolate [17, 18]. Folic acid is an oxidized and stable synthetic form of folate that can only be obtained from fortified food, supplements, and pharmaceuticals [5]. Its chemical structure is illustrated in Fig. 1.Both natural folates and folic acid are converted into 5-mTHF, the final product of the folate cycle, which is the principal active metabolite of folate. Its stable calcium salt is widely used for pharmaceutical use [19].

**Fig. 2:**
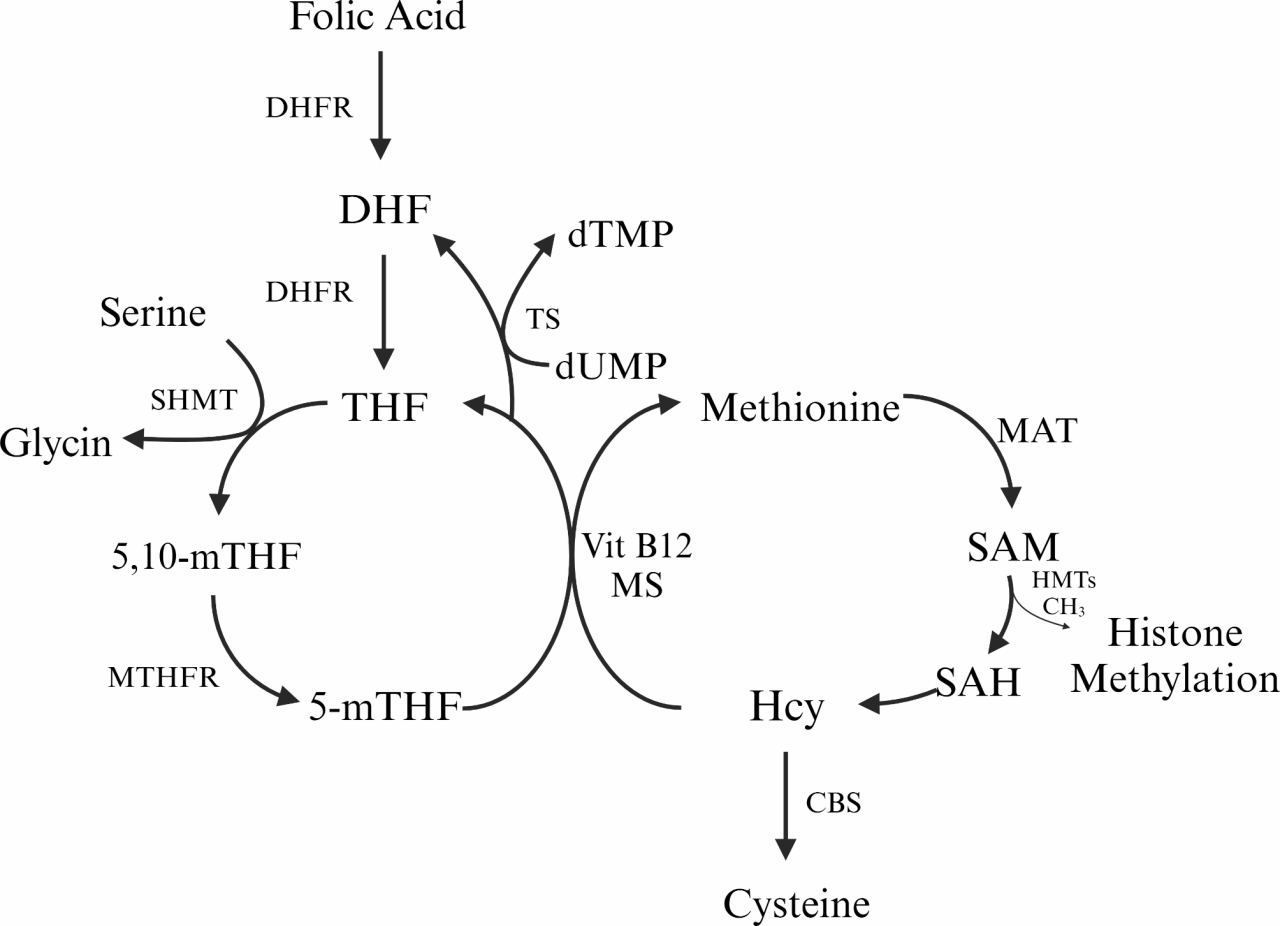
Main metabolic pathways of folate

Food folates (polyglutamate derivatives) are hydrolyzed at the mucosal epithelial cell brush border by folyl-poly-glutamate carboxypeptidase to the monoglutamate form [20]. Then, both food folates and folic acid are absorbed in the proximal small intestine (jejunum) via the proton-coupled folate transporter [21]. In mucosal cells, folic acid is reduced to tetrahydrofolate (THF) by dihydrofolate reductase (DHFR). (Fig. 2) Then, THF is metabolized to 5-mTHF with the aid of serine hydroxymethyltransferase and 5,10-methylenetetrahydrofolate reductase (MTHFR) [20]. 5-mTHF is both an active and final product of folate in the body, which accounts for approximately 98% of folates in human plasma [5]. After absorption, it is released into the portal circulation, most of it is taken up by the liver, and the rest of it is secreted into the gut by the bile duct and recirculated by the enterohepatic cycle [22]. Endogenous folate exists in free and combined forms in plasma [1].

Folate has an important role in one-carbon metabolism, which primarily involves the metabolism of serine and histidine and the synthesis of thymidylate, methionine, and purine [1]. The critical components of one-carbon metabolism are the folate and methionine cycles. THF is an intermediate carrier of a single carbon unit because of its unique chemical structure with the presence of a pterin core, which has π-electron-deficient properties and makes it easy to accept a carbon unit [19, 23]. With the help of serine hydroxymethyltransferase, THF forms 5,10-methylene-THF by accepting a single carbon group from serine and generating glycine at the same time. Afterward, 5,10-methylene-THF participates in the formation of deoxythymidine monophosphate (dTMP) and dihydrofolate (DHF) by reducing and methylating deoxyuridine monophosphate, catalyzed by the thymidylate synthase enzyme [24]. dTMP is next involved in the synthesis of DNA. DHF can re-enter the folate cycle and be reduced to THF. In the methionine cycle, methionine generates S-adenosylmethionine (SAM) via the adenylation of methionine by methionine adenosyltransferase. SAM, as the methyl donor, donates a methyl group to the histone and generates S-adenosylhomocysteine (SAH) by histone methyltransferases (HMTs). Then, SAH further generates homocysteine. Homocysteine is eliminated by producing cysteine via Cystathionine-beta-synthase (CBS) **(**Fig. 2). Homocysteine is the intermediate product of the methionine cycle and can be converted back to methionine by donation of a methyl group from 5-mTHF or betaine [25]. Therefore, the complete methionine cycle needs the assistance of folate, and folate deficiency can impair the remethylation of homocysteine and cause the accumulation of homocysteine and abnormal one-carbon metabolism. From another aspect, plasma homocysteine concentration is an indirect indicator of folate level [26].

### Folate and retinal vascular occlusions

Retinal vascular occlusions are common causes of visual loss. Retinal vascular occlusions can be divided into two main types: retinal artery occlusion (RAO) and retinal vein occlusion (RVO). These two main types of occlusions have different etiologies, pathogeneses, and clinical features. However, both are closely related to aging and cardiovascular risk factors. RVO is more prevalent than RAO and seems to have much better endpoints [27]. Retinal vascular occlusion can be divided into the central vascular occlusion type and branch vascular occlusion type according to the scope of occlusion.

Homocysteine, a sulfur-containing amino acid, is an intermediate product of methionine metabolism. Elevated plasma homocysteine levels, a prothrombotic state, are an accepted risk factor for atherosclerosis and cardiovascular diseases [28]. Hyperhomocysteinemia is defined as a fasting total plasma homocysteine level greater than 15 µmol/L. Homocysteine has been considered to injure vascular endothelial cells by impairing the endothelium’s capacity, increasing pro-thrombotic platelet function, inducing the production of pro-inflammatory cytokines, and mediating the apoptotic cell death of endothelial cells [29]. Hyperhomocysteinemia will sustain a snowball-like effect and irreversibly impair endothelial cells. The causes of hyperhomocysteinemia include dietary deficiencies of the vitamin B complex, genetic defects such as a thermolabile variant of MTHFR, clinical conditions such as renal failure, and some drugs, such as methotrexate and theophylline [30].

Many clinical studies have reported that plasma homocysteine levels are significantly higher in patients with retinal vascular occlusive disease and have suggested that hyperhomocysteinemia is a risk factor for retinal vascular occlusion disease [9, 31–34]. Some case-control studies found that the plasma homocysteine level in the patient group was significantly higher than that in the control group and assumed that hyperhomocysteinemia was a risk factor for both RAO and RVO diseases [14, 35–40]. Table 1 summarizes clinical trials investigating the associations between homocysteine as well as folate and RAO, RVO. The most common cause of retinal artery disease is embolism, which mostly comes from the carotid artery and the heart [41]. It was found that hyperhomocysteinemia, as a recognized risk factor for thrombosis, was associated with an increased incidence of retinal emboli in an older population-based cross-sectional study [42]. The association between hyperhomocysteinemia and RAO disease was confirmed by a meta-analysis of cohort studies [43]. In the aforementioned studies, folate was used to reduce plasma homocysteine concentrations.

**Table 1:** Clinical trials investigating the associations between homocysteine and folate and RAO and RVO

Source	Study type	NO. of pts	Mean age (yrs)	Mean folate (ng/dl)	P value	Mean homocysteine (µmol/l)	P value
***RAO*** Pianka et al. (2000) [37]	Case-control	13	69.38	N/A	N/A	12.5	0.0034
Weger et al. (2002) [36]	Case-control	105	69.1	5.6	0.040	12.2	0.003
Narin et al. (2004) [35]	Case-control	20	55.8	N/A	N/A	21.23	< 0.08
***RVO*** Weger et al. (2002) [44]	Case-control	84	68.1	4.5	0.007	11.4	0.002
Yildirim et al. (2004) [33]	Case-control	23	61	6.3	0.82	11.7	0.005
Gao et al. (2006) [14]	Case-control	64	59.5	5.62	0.032	13.83	0.003
Sofi et al. (2008) [39]	Case-control	262	66	5.5	< 0.0001	12.4	< 0.0001
Napal et al. (2021) [38]	Case-control	368	67.9	7	< 0.001	13.4	< 0.0001

Likewise, a review reported that hyperhomocysteinemia could increase central retinal vein occlusion (CRVO) risk and is a critical factor in the development of CRVO [45]. A meta-analysis demonstrated that retinal vascular occlusion was significantly correlated with elevated plasma total homocysteine levels and low serum folate levels [46]. Folate supplementation was used to lower the plasma hyperhomocysteine concentration by converting hyperhomocysteine to methionine. Initially, the benefit of folic acid on RVO was considered to be the result of a reduction in plasma total homocysteine concentrations. However, many studies have found that a large number of RVO patients, especially CRVO patients, always have elevated plasma homocysteine levels and low plasma folate levels. Therefore, many scholars assume that low folate status is an independent risk factor for CRVO. Some case-control studies were conducted to determine the relationship between folate status and CRVO. Gao et al. [14] reported an odds ratio (OR) of 1.3 for plasma folate in patients with CRVO in the Chinese population, and an OR of 6.13 (95% confidence interval (CI) 3.85–9.76, P < 0.0001) was reported by another study conducted in Florence [39]. Another case-control study found that RVO patients had lower serum folate levels than population-based controls of similar age and sex (P < 0.0001) and suggested that treatment with folate supplementation may be useful [38]. A meta-analysis also found the plasma folate level was significantly lower in RVO patients compared with controls (P = 0.001), as well as in CRVO patients (P = 0.006), and indicated that low serum folate levels were a risk factor of RVO [38]. A study even suggested that any patients with elevated homocysteine levels should be treated with folic acid supplementation [47]. Availble studies suggested that hyperhomocysteinmia was an risk factors for RVO and RAO. Compared with RVO, only a few researchers have aimed to investigate the association between hyperhomocysteinemia and RAO. This seems to be due to the lower incidence of RAO than RVO. Moreover, because of the small sample size and amount of research, more studies are needed to confirm the association between hyperhomocysteinemia and RAO. .

In some cases, retinal vascular occlusive disease occurs in young adults with no other systemic disease or comorbidity but a high homocysteine status [48–50]. In some case reports, RAO occurred in young patients with a homozygous MTHFR genetic defect [51, 52]. Interestingly, another case report presented a 12-year-old girl with CRVO, and the existence of a homozygous C677T MTHFR mutation emerged in retinal neovascularization twelve years after the first treatment. Folate deficiency with the existence of an MTHFR genetic defect could cause hyperhomocysteinemia and then accelerate atherosclerosis or thrombosis and even neovascularization [53]. Folate supplementation should be considered in those patients who have MTHFR genetic defects accompanied by folate deficiency. Folic acid is essential for cell survival and growth. Folic acid also has several beneficial effects on vascular endothelial cells in addition to reducing homocysteine-induced toxic effects by lowering plasma homocysteine levels. Folic acid can protect human umbilical vein endothelial cells (HUVECs) from injury caused by hypoxia by targeting the ERK1/2/NOX/ROS pathway [54]. Another study found that folic acid can inhibit cell apoptosis and reduce homocysteine-induced toxicity by regulating the expression of apoptosis-related genes in HUVECs [55]. These studies illustrate the function of folic acid in HUVECs, and its effect on ocular needs to be assessed by more studies.

### Folate and diabetic retinopathy

DR is the leading cause of visual impairment in working-age people [56]. DR is a specific microvascular complication of diabetes mellitus (DM) characterized by functional and morphological changes in the retina. DR can be classified into nonproliferative DR and proliferative DR according to the presence of neovascularization. As the most advanced stage of DR, proliferative DR is characterized by neovascularization and proliferative membrane formation, which may cause vitreous hemorrhage and tractional retinal detachment, resulting in progressive vision loss. The mechanism of DR pathogenesis is diverse and complicated, including activation of inflammatory pathways, endoplasmic reticulum (ER) stress, reactive oxygen species production, overexpression of vascular endothelial growth factor, and genetic and epigenetic factors [57].

Since homocysteine was found to have toxic effects on vascular endothelial cells, the association between homocysteine and the prevalence of retinopathy was investigated. Some patients with diabetes were also observed to have hyperhomocysteinemia. However, the association between retinopathy and homocysteine is controversial. Retinopathy, similar to microangiopathy, was initially not found to be associated with elevated plasma homocysteine concentrations [58–60]. However, unlike these inconsistent results, proliferative DR was observed to be related to homocysteine concentrations in type 2 diabetes mellitus (T2DM) [61]. Some observations supported that elevated plasma homocysteine levels were an independent risk factor for the development and progression of DR in T2DM [13, 62]. Recently, a research team focused on homocysteine’s role in human visual disorders, especially DR and other age-related diseases. They found that hyperhomocysteinemia breaks up inner and outer blood-retinal barriers, induces retinal ischemia and neovascularization, activates oxidative and ER stresses, and induces epigenetic modifications [11, 63–65]. They found that the underlying mechanism of these pathologies induced by hyperhomocysteinemia is inflammation, which is mediated by NF-κB activation and regulation of inflammation-related cytokines [66]. Hence, they suggested that homocysteine may be a potential biological marker and a therapeutic target for DR [66, 67].

The concentration of plasma homocysteine is always associated with the status of B vitamins. A study analyzed the data from eligible diabetic patients from the US National Health and Nutrition Examination Survey from 2003 to 2018 and found that dietary folate intake could decrease the risk of DR [68]. Metanx, a prescription product, contains L-methylfolate calcium, which is the active form of vitamins B9 and other essential B vitamins (B6 and B12) [69]. It has been used to relieve the symptoms of diabetic neuropathy in diabetic patients. In recent decades, metanx was found to have positive impacts on endothelial dysfunction [70–73]. A pilot study found that metanx treatment seemed to help reduce retinal edema and increase light sensitivity in patients with nonproliferative DR [74]. An animal experiment showed that metanx inhibited measures of oxidative stress and inflammation and might assist in inhibiting diabetes-induced defects in visual function [69]. A meta-analysis found that folic acid supplementation may reduce total homocysteine levels in T2DM patients and has a trend to be associated with better glycemic control compared with placebo [75]. In addition to vitamin supplementation, there are also many clinical studies exploring the relationship between folate status and DM patients with or without DR. Fotiou et al. [13]found that patients with DR had significantly lower serum folate (P < 0.001) than those without DR in T2DM, and plasma levels of folate and vitamin B12, as the determinants of serum homocysteine concentration, may also affect DR risk. Moreover, they suggested that monitoring plasma folate levels could be used as an indicator for assessing the risk of DR. A study found that T2DM patients with proliferative DR and nonproliferative DR had significantly low folate status [76]. Folic acid may play a role in the development and progression of DR. Folic acid was found to protect retinal vascular endothelial cells against high glucose-induced injury from several aspects. Folic acid can diminish the expression of YAP1 and TEAD1 in the Hippo signaling pathway. The Hippo signaling pathway is associated with angiogenesis, which is the major pathological change in DR [77]. Furthermore, an animal study found that folic acid could protect the retina from thinning in the early stage of DR in diabetes mouse models by downregulating a panel of genes associated with angiogenesis, inflammation, and oxidative stress [78]. Folic acid could regulate the metabolism of DNA methylation, a kind of epigenetic change, in retinal microvessel cells against high glucose [79]. These experimental findings illustrate the possible mechanism of the protective effect of folic acid in DR. More studies still need to be conducted to confirm the association between folic acid and DR and the effect of folic acid supplementation.

### Folate and other retinal vascular diseases

As mentioned above, homocysteine has been recognized as a risk factor for cardiovascular diseases and age-related diseases. Age-related macular degeneration (AMD), which affects photoreceptors, retinal pigment epithelium (RPE), Bruch’s membrane, and the choroid complex, is the leading cause of irreversible visual impairment, accounting for 6–9% of global blindness [80]. The mechanism of folate against AMD might be through the prevention of elevated serum homocysteine by promoting the elimination of homocysteine [81]. Some studies reported that the plasma concentration of homocysteine was significantly increased in AMD patients compared with the control group [82–85]. A cross-sectional analysis found that patients with late-stage AMD had significantly lower folate intakes compared with controls (P < 0.0001) and folate had no statistically significant association with AMD [86]. Nevertheless, several observational studies reported that serum folate was significantly associated with AMD risk [84, 87, 88]. A post hoc analysis of two age-related eye disease studies found that higher dietary intake of multiple nutrients including folate was associated with decreased risk of progression to late-stage AMD [89]. Some studies also suggested that a healthy diet rich in B vitamins, including folic acid, might reduce the risk of AMD and vision loss due to AMD [84, 87]. The Mediterranean diet, a diet with large consumption of vegetables, fruits, and nuts, is rich in B vitamins and minerals and can be recommended. More studies are needed to determine the effects of folic acid on AMD.

Bilateral retinal hemorrhages were observed in a 33-year-old man with macrocytic anemia and dietary folate deficiency in a case report. They assumed that retinal hemorrhage was a complication of the hyperdynamic retinal circulation and tissue hypoxia induced by severe macrocytic anemia, which seemed to be the result of folate deficiency [90]. A study observed significantly increased cerebrospinal folate levels in cerebral malaria patients after effective treatment of the acute malaria phase [91]. A comment suggested that folate deficiency might participate in the mechanism of retinal hemorrhages in cerebral malaria [92]. To date, its specific mechanism remains unclear.

### Folate Supplementation

External folate supplementation includes folic acid, L-methylfolate, folinic acid, etc. Mandatory folic acid fortification of grain products has been used in certain countries, such as Canada, and has succeeded in decreasing the rates of neural tube defects by 20%~50%. Folic acid supplementation has been proven to reduce the risk of neural tube defects [93, 94]. It is widely used to treat hyperhomocysteinemia and folate deficiency. It has been shown that daily folic acid supplementation of 0.5-5.0 mg folic acid could reduce plasma homocysteine levels by approximately a quarter to a third, typically in the western population [15, 95]. However, long-term consumption of folic acid supplementation is controversial. Due to the improvement in diet and volunteer intake of fortified food and supplements, some people may ingest excess folate (upper limit: 1 mg). In 2015, the WHO defined high folate status as plasma folate greater than 45.3 nmol/L [96]. The imbalance of high folate status and low vitamin B12 could predispose pregnant women to diabetes and their offspring to insulin resistance [97]. High folic acid intake may increase the risk of certain types of cancer, including colorectal cancer and prostate cancer [98, 99]. Numerous studies have been conducted to investigate the controversial effect of folic acid on cancer risk. Some studies conclude that folic acid supplementation will not influence overall and site-specific cancer incidence [100, 101]. This controversy may be relevant to folate status, cancer stage, different baseline information, etc. More studies are still needed to explore the effect of folic acid supplementation on cancer risk. For retinal vascular diseases, a systematic review indicated that folic acid supplements (2.5 mg) could not prevent AMD and increased the risk of development of AMD. [102]. Another concern is that a high intake of folic acid may mask the appearance of vitamin B12 anemia and may interact with DHFR inhibitors such as methotrexate, making it less effective [5, 103]. A pilot study showed that a three-month vitamin supplementation containing L-methylfolate could significantly reduce plasma homocysteine levels in patients with diabetes [104]. L-methylfolate is as effective as folic acid in improving folate status and less likely to mask the appearance of vitamin B12-induced anemia and interact with DHFR inhibitors. Although folate supplementation could effectively reduce plasma homocysteine levels, its roles in the reduction of intracellular homocysteine toxicity, the therapeutic effect, and the prevention of retinal vascular diseases remain uncertain. Moreover, it may even increase the risk of development of AMD or certain types of cancer. It can not be recommended unless the clear evidence of benefit is needed.

## Discussion

**Fig. 3:**
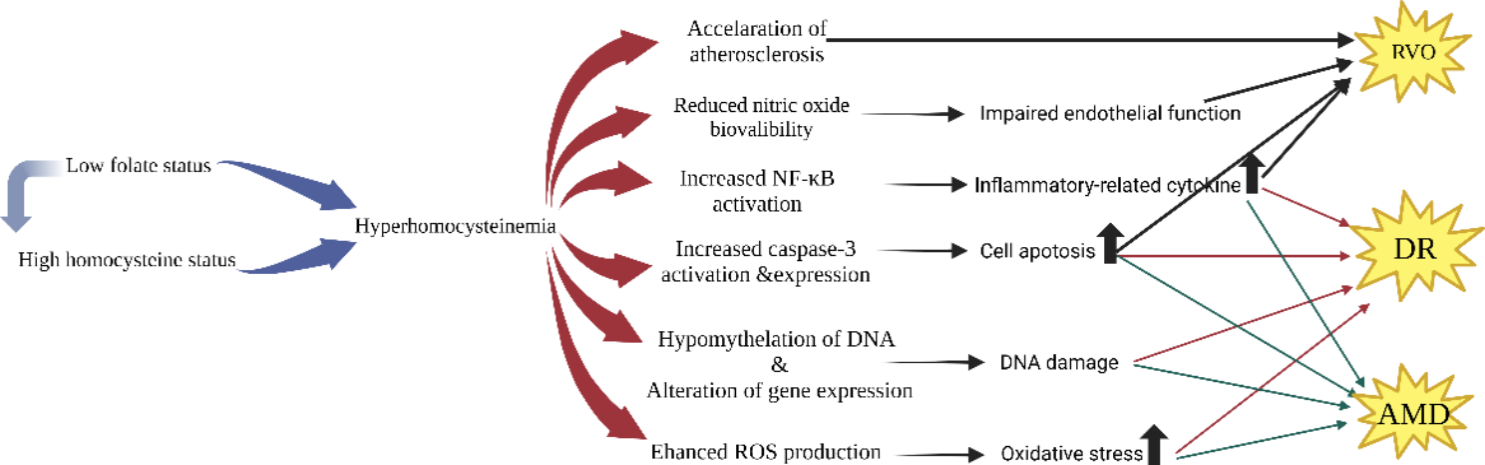
Pathophysiological changes caused by hyperhomocysteinemia

High plasma homocysteine levels are independent risk factors for RVO, which is essentially associated with major vascular factors and is considered as a form of atherosclerosis. Hyperhomocysteinemia, as a prothrombotic state, is a recognized risk factor for atherosclerosis and is usually the consequence of folate deficiency and, to a lesser extent, vitamin B12 deficiency. According to the available studies, both low plasma folate levels and high plasma homocysteine levels are independent risk factors for RVO. For AMD and DR, hyperhomocysteinmia has been considered as an independent risk factor. And yet it is still not certain that folic acid is an independent risk factor for AMD, only that it is associated with disease progression. These studies do not show an internal relationship between them. Retinal vasculopathy due to low folate status, which is essentially high homocysteine endangering the retinal vascular. Moreover, folic acid supplementation can effectively reduce and control plasma homocysteine levels and relief the high homocysteine-induced damage. The pathophysiologic changes caused by hyperhomocysteinemia are illustrated in Fig. 3. These pathways could be therapeutic targets and the role of folate stills needs more exploration. A systematic review reported that folic acid supplementation may improve endothelia function by increasing flow-mediated dilation and the percentage of flow-mediated dilation. The intrinsic mechanism is to improve the bioavailability of nitric oxide and to act as a direct scavenger of oxygen species, both of which are mediated independently of its homocysteine-lowering effect. [105, 106] Folate is essential for many important process such as cells growth and cell proliferation. Several animal studies suggested that folic acid can suppress on angiogenesis, inflammation and oxidative stress through epigenetic regulation to counteract diabetic retinopathy progression. [78, 79] In addition to protecting endothelial cells from homocysteine-induced toxic effects, folate has been found that it could inhibit cell apoptosis by regulating the expression of apoptosis-related genes in HUVECs. Hence, folate may have a protective effect against retinal vascular diseases. More prospective clinical studies are needed to explore the effect of folic acid supplementation on protection and prevention.

## Conclusion

Folate, as a critical cofactor of the one-carbon cycle, can reduce the serum homocysteine level via methionine metabolism. Some studies have suggested that hyperhomocysteinemia is independent risk factors for RVO, AMD and nonproliferative DR (in T2DM). Folate status is associated with the severity of DR and progression of AMD. The present review provides evidence that dietary folate intake is thought to have a protective effect on some ocular vascular diseases that are damaged by hyperhomocysteinemia and high glucose. For its effect on the treatment and prevention of diseases, there is still insufficient and uniform evidence. Therefore, further studies are required to assess the effect of folate or its supplementation on retinal vascular diseases in a large population.

## Data Availability

Data availability is not applicable to this article as no new data were created or analyzed in this study.

## Abbreviations

RVO: Retinal vascular occlusion
CRVO: Central retinal vein occlusion
CRAO: Central retinal artery occlusion
DR: Diabetes retinopathy
AMD: Age-related macular degeneration
DHFR: Dihydrofolate Reductase
T2DM: Type 2 diabetes mellitus
THF: Tetrahydrofolate
5,10-mTHF: 5, 10-methyl-Tetrahydrofolate
5-mTHF: 5-methyltetrahydrofolate
SHMT: Serine hydroxymethyltransferase
MTHFR: Methylenetetrahydrofolate Reductase
TS: Thymidylate synthase
dTMP: Deoxythymidine monophosphate
dUMP: Deoxyuridine monophosphate
MAT: Methionine adenosyltransferase
SAM: S-Adenosylmethionine
SAH: S-adenosylhomocysteine
HMTs: Histonemethyltransferases
Hcy: Homocysteine
MS: Methionine synthase
CBS: Cystathionine-beta-synthase
HUVECs: human umbilical vein endothelial cells
ER: Endoplasmic reticulum
RPE: retinal pigment epithelium

## References

[1] Lucock M (2000). Folic acid: nutritional biochemistry, molecular biology, and role in disease processes. Mol Genet Metab.

[2] Gregory JF (2001). 3rd, *Case study: folate bioavailability*. J Nutr.

[3] Wills L. *Treatment of “pernicious anaemia of pregnancy” and “tropical anaemia” with special reference to yeast extract as a curative agent. 1931* Nutrition, 1991. 7(5): p. 323-7; discussion 328. 1804466

[4] Pitkin RM (2007). Folate and neural tube defects. Am J Clin Nutr.

[5] Pietrzik K, Bailey L, Shane B (2010). Folic acid and L-5-methyltetrahydrofolate: comparison of clinical pharmacokinetics and pharmacodynamics. Clin Pharmacokinet.

[6] Blount BC (1997). Folate deficiency causes uracil misincorporation into human DNA and chromosome breakage: implications for cancer and neuronal damage. Proc Natl Acad Sci U S A.

[7] Ganguly P, Alam SF (2015). Role of homocysteine in the development of cardiovascular disease. Nutr J.

[8] Seshadri S (2006). Elevated plasma homocysteine levels: risk factor or risk marker for the development of dementia and Alzheimer’s disease?. J Alzheimers Dis.

[9] Cahill M (2000). Raised plasma homocysteine as a risk factor for retinal vascular occlusive disease. Br J Ophthalmol.

[10] Hankey GJ, Eikelboom JW (2001). Homocysteine and stroke. Curr Opin Neurol.

[11] Elmasry K (2018). Epigenetic modifications in hyperhomocysteinemia: potential role in diabetic retinopathy and age-related macular degeneration. Oncotarget.

[12] Bek T (2013). Regional morphology and pathophysiology of retinal vascular disease. Prog Retin Eye Res.

[13] Fotiou P et al. *Vitamin status as a determinant of serum homocysteine concentration in type 2 diabetic retinopathy* J Diabetes Res, 2014. 2014: p. 807209. 10.1155/2014/807209 PMC4071945 25006590

[14] Gao W (2006). Hyperhomocysteinemia and low plasma folate as risk factors for central retinal vein occlusion: a case-control study in a chinese population. Graefes Arch Clin Exp Ophthalmol.

[15] Liakishev AA (2006). [Homocysteine lowering with folic acid and B vitamins in vascular disease]. Kardiologiia.

[16] Scott JM, Weir DG (1976). Folate composition, synthesis and function in natural materials. Clin Haematol.

[17] Wright AD, Martin N, Dodson PM (2008). Homocysteine, folates, and the eye. Eye (Lond).

[18] Thien KR (1977). Serum folates in man. J Clin Pathol.

[19] Shulpekova Y et al. *The Concept of Folic Acid in Health and Disease*. Molecules, 2021. 26(12). 10.3390/molecules26123731 PMC8235569 34207319

[20] Chandler CJ, Wang TT, Halsted CH (1986). Pteroylpolyglutamate hydrolase from human jejunal brush borders. Purification and characterization. J Biol Chem.

[21] Zhao R, Matherly LH, Goldman ID (2009). Membrane transporters and folate homeostasis: intestinal absorption and transport into systemic compartments and tissues. Expert Rev Mol Med.

[22] Wright AJ, Dainty JR, Finglas PM (2007). Folic acid metabolism in human subjects revisited: potential implications for proposed mandatory folic acid fortification in the UK. Br J Nutr.

[23] Relton CL (2004). Low erythrocyte folate status and polymorphic variation in folate-related genes are associated with risk of neural tube defect pregnancy. Mol Genet Metab.

[24] Locasale JW (2013). Serine, glycine and one-carbon units: cancer metabolism in full circle. Nat Rev Cancer.

[25] Mentch SJ, Locasale JW (2016). One-carbon metabolism and epigenetics: understanding the specificity. Ann N Y Acad Sci.

[26] Blom HJ, Smulders Y (2011). Overview of homocysteine and folate metabolism. With special references to cardiovascular disease and neural tube defects. J Inherit Metab Dis.

[27] Scott IU (2020). Retinal vascular occlusions. Lancet.

[28] Boushey CJ (1995). A quantitative assessment of plasma homocysteine as a risk factor for vascular disease. Probable benefits of increasing folic acid intakes. JAMA.

[29] Esse R et al. *The contribution of Homocysteine Metabolism disruption to endothelial dysfunction: state-of-the-art*. Int J Mol Sci, 2019. 20(4). 10.3390/ijms20040867 PMC6412520 30781581

[30] Ueland PM (1993). Total homocysteine in plasma or serum: methods and clinical applications. Clin Chem.

[31] Martin SC (2000). Plasma total homocysteine and retinal vascular disease. Eye (Lond).

[32] Abu El-Asrar AM (2002). Hyperhomocysteinemia and retinal vascular occlusive disease. Eur J Ophthalmol.

[33] Yildirim C (2004). Hyperhomocysteinemia: a risk factor for retinal vein occlusion. Ophthalmologica.

[34] Sottilotta G (2007). Role of hyperhomocystinemia in retinal vascular occlusive disease. Clin Appl Thromb Hemost.

[35] Narin F (2004). Plasma homocysteine and retinal artery occlusive disease: a case-control study. Ann Saudi Med.

[36] Weger M (2002). The role of hyperhomocysteinemia and methylenetetrahydrofolate reductase (MTHFR) C677T mutation in patients with retinal artery occlusion. Am J Ophthalmol.

[37] Pianka P (2000). Hyperhomocystinemia in patients with nonarteritic anterior ischemic optic neuropathy, central retinal artery occlusion, and central retinal vein occlusion. Ophthalmology.

[38] Napal Lecumberri JJ (2021). Lipid profile and serum folate, vitamin B(12) and homocysteine levels in patients with retinal vein occlusion. Clin Investig Arterioscler.

[39] Sofi F (2008). Low vitamin B6 and folic acid levels are associated with retinal vein occlusion independently of homocysteine levels. Atherosclerosis.

[40] Weger M (2002). Hyperhomocyst(e)inemia, but not methylenetetrahydrofolate reductase C677T mutation, as a risk factor in branch retinal vein occlusion. Ophthalmology.

[41] Hayreh SS, Podhajsky PA, Zimmerman MB (2009). Retinal artery occlusion: associated systemic and ophthalmic abnormalities. Ophthalmology.

[42] Chua B (2006). Homocysteine and retinal emboli: the Blue Mountains Eye Study. Am J Ophthalmol.

[43] Huang X (2017). Homocysteine in retinal artery occlusive disease: a meta-analysis of cohort studies. Sci Rep.

[44] Weger M (2002). Hyperhomocyst(e)inemia and MTHFR C677T genotypes in patients with central retinal vein occlusion. Graefes Arch Clin Exp Ophthalmol.

[45] Janssen MC (2005). Retinal vein occlusion: a form of venous thrombosis or a complication of atherosclerosis? A meta-analysis of thrombophilic factors. Thromb Haemost.

[46] Cahill MT, Stinnett SS, Fekrat S (2003). Meta-analysis of plasma homocysteine, serum folate, serum vitamin B(12), and thermolabile MTHFR genotype as risk factors for retinal vascular occlusive disease. Am J Ophthalmol.

[47] Kaye AD (2020). Folic acid supplementation in patients with elevated homocysteine levels. Adv Ther.

[48] Liu Z (2019). Central retinal venous occlusion in a child with hyperhomocysteinemia: a case report. Med (Baltim).

[49] Zahid S (2020). Homocystinuria in a 14-year old girl manifesting as central retinal artery occlusion: a case report. J Pak Med Assoc.

[50] Taubert M, Dowd TC, Wood A (2008). Malnutrition and bilateral central retinal vein occlusion in a young woman: a case report. J Med Case Rep.

[51] Shute C (2018). A case report of branch retinal artery occlusion in a teenager due to hyperhomocysteinaemia; the interplay of genetic and nutritional defects. BMC Ophthalmol.

[52] Coban-Karatas M (2013). Central retinal artery occlusion in a 13-year-old child as a presenting sign of hyperhomocysteinemia together with high lipoprotein(a) level. Pediatr Neurol.

[53] Hudson JL, Laura DM, Berrocal AM. Central retinal vein occlusion in 12-year-old girl with Methylenetetrahydrofolate reductase mutation: a Case Report and Review of the literature. Retin Cases Brief Rep; 2022. 10.1097/ICB.0000000000001283 35385432

[54] Cheng F (2016). Folic acid attenuates vascular endothelial cell Injury caused by Hypoxia via the inhibition of ERK1/2/NOX4/ROS pathway. Cell Biochem Biophys.

[55] Cui S (2018). Folic acid inhibits homocysteine-induced cell apoptosis in human umbilical vein endothelial cells. Mol Cell Biochem.

[56] Ogurtsova K (2017). IDF Diabetes Atlas: global estimates for the prevalence of diabetes for 2015 and 2040. Diabetes Res Clin Pract.

[57] Solanki A (2019). Targeting Matrix Metalloproteinases for Diabetic Retinopathy: the way ahead?. Curr Protein Pept Sci.

[58] Agardh CD (1994). Lack of association between plasma homocysteine levels and microangiopathy in type 1 diabetes mellitus. Scand J Clin Lab Invest.

[59] Smulders YM (1999). Fasting and post-methionine homocysteine levels in NIDDM. Determinants and correlations with retinopathy, albuminuria, and cardiovascular disease. Diabetes Care.

[60] Buysschaert M (2000). Hyperhomocysteinemia in type 2 diabetes: relationship to macroangiopathy, nephropathy, and insulin resistance. Diabetes Care.

[61] Looker HC (2003). Homocysteine as a risk factor for nephropathy and retinopathy in type 2 diabetes. Diabetologia.

[62] Brazionis L (2008). Homocysteine and diabetic retinopathy. Diabetes Care.

[63] Ibrahim AS (2016). Hyperhomocysteinemia disrupts retinal pigment epithelial structure and function with features of age-related macular degeneration. Oncotarget.

[64] Tawfik A, Smith SB (2014). Increased ER stress as a mechanism of retinal neurovasculopathy in mice with severe hyperhomocysteinemia. Austin J Clin Ophthalmol.

[65] Tawfik A (2013). Alterations of retinal vasculature in cystathionine-Beta-synthase mutant mice, a model of hyperhomocysteinemia. Invest Ophthalmol Vis Sci.

[66] Elsherbiny NM et al. *Homocysteine induces inflammation in retina and brain*. Biomolecules, 2020. 10(3). 10.3390/biom10030393 PMC7175372 32138265

[67] Tawfik A et al. *Homocysteine: a potential biomarker for Diabetic Retinopathy*. J Clin Med, 2019. 8(1). 10.3390/jcm8010121 PMC6352029 30669482

[68] Zhang G et al. *Certain Dietary Nutrients Reduce the Risk of Eye Affliction/Retinopathy in Individuals with Diabetes: National Health and Nutrition Examination Survey, 2003–2018* Int J Environ Res Public Health, 2022. 19(19). 10.3390/ijerph191912173 PMC9566346 36231475

[69] Liu H (2015). Metanx and early stages of diabetic retinopathy. Invest Ophthalmol Vis Sci.

[70] Walker MJ, Morris LM, Cheng D (2010). Improvement of cutaneous sensitivity in diabetic peripheral neuropathy with combination L-methylfolate, methylcobalamin, and pyridoxal 5’-phosphate. Rev Neurol Dis.

[71] Jacobs AM, Cheng D (2011). Management of diabetic small-fiber neuropathy with combination L-methylfolate, methylcobalamin, and pyridoxal 5’-phosphate. Rev Neurol Dis.

[72] Shevalye H (2012). Metanx alleviates multiple manifestations of peripheral neuropathy and increases intraepidermal nerve fiber density in Zucker diabetic fatty rats. Diabetes.

[73] Fonseca VA (2013). Metanx in type 2 diabetes with peripheral neuropathy: a randomized trial. Am J Med.

[74] Smolek MK (2013). Intervention with vitamins in patients with nonproliferative diabetic retinopathy: a pilot study. Clin Ophthalmol.

[75] Sudchada P (2012). Effect of folic acid supplementation on plasma total homocysteine levels and glycemic control in patients with type 2 diabetes: a systematic review and meta-analysis. Diabetes Res Clin Pract.

[76] Malaguarnera G (2015). Folate status in type 2 diabetic patients with and without retinopathy. Clin Ophthalmol.

[77] Wang Z et al. *Folic acid has a protective effect on retinal vascular endothelial cells against high glucose*. Molecules, 2018. 23(9). 10.3390/molecules23092326 PMC6225375 30213067

[78] Lei XW (2019). The protective roles of folic acid in preventing Diabetic Retinopathy are potentially Associated with Suppressions on Angiogenesis, inflammation, and oxidative stress. Ophthalmic Res.

[79] Liu X, Cui H (2021). The palliative effects of folic acid on retinal microvessels in diabetic retinopathy via regulating the metabolism of DNA methylation and hydroxymethylation. Bioengineered.

[80] Wong WL (2014). Global prevalence of age-related macular degeneration and disease burden projection for 2020 and 2040: a systematic review and meta-analysis. Lancet Glob Health.

[81] Christen WG (2018). Prospective study of plasma homocysteine, its dietary determinants, and risk of age-related macular degeneration in men. Ophthalmic Epidemiol.

[82] Nowak M (2004). [Blood concentration of homocysteine, vitamin B (12), and folic acid in patients with exudative age related macular degeneration]. Klin Oczna.

[83] Kamburoglu G (2006). Plasma homocysteine, vitamin B12 and folate levels in age-related macular degeneration. Graefes Arch Clin Exp Ophthalmol.

[84] Gopinath B (2013). Homocysteine, folate, vitamin B-12, and 10-y incidence of age-related macular degeneration. Am J Clin Nutr.

[85] Nowak M (2005). Homocysteine, vitamin B12, and folic acid in age-related macular degeneration. Eur J Ophthalmol.

[86] Rochtchina E (2007). Elevated serum homocysteine, low serum vitamin B12, folate, and age-related macular degeneration: the Blue Mountains Eye Study. Am J Ophthalmol.

[87] Merle BMJ et al. *B vitamins and incidence of Advanced Age-Related Macular Degeneration: the Alienor Study*. Nutrients, 2022. 14(14). 10.3390/nu14142821 PMC9318446 35889778

[88] Nunes S (2018). Adherence to a Mediterranean diet and its association with age-related macular degeneration. The Coimbra Eye Study-Report 4. Nutrition.

[89] Agrón E (2021). Dietary nutrient intake and progression to late age-related Macular Degeneration in the Age-Related Eye Disease Studies 1 and 2. Ophthalmology.

[90] Hughes M, Leach M (2006). Dietary folate deficiency and bilateral retinal haemorrhages. Lancet.

[91] Areekul S, Cheeramakara C (1985). Cerebrospinal fluid folate activity in patients with Plasmodium falciparum cerebral malaria. Trop Geogr Med.

[92] Eisenhut M. *Role of folate deficiency in the pathogenesis of retinal and cerebral hemorrhages in cerebral malaria* Am J Trop Med Hyg, 2007. 76(5): p. 793; author reply 793-4. 17488892

[93] Czeizel AE (2013). Folate deficiency and folic acid supplementation: the prevention of neural-tube defects and congenital heart defects. Nutrients.

[94] Ray JG (2004). Folic acid food fortification in Canada. Nutr Rev.

[95] Collaboration HLT (1998). Lowering blood homocysteine with folic acid based supplements: meta-analysis of randomised trials. Homocysteine lowering Trialists’ collaboration. BMJ.

[96] WHO. Serum and red blood cell folate concentrations for assessing folate status in populations. Vitamin and Mineral Nutrition Information System; 2015.

[97] Paul L, Selhub J (2017). Interaction between excess folate and low vitamin B12 status. Mol Aspects Med.

[98] Oliai Araghi S (2019). Folic acid and vitamin B12 supplementation and the risk of Cancer: long-term follow-up of the B vitamins for the Prevention of osteoporotic fractures (B-PROOF) trial. Cancer Epidemiol Biomarkers Prev.

[99] Wien TN (2012). Cancer risk with folic acid supplements: a systematic review and meta-analysis. BMJ Open.

[100] Vollset SE (2013). Effects of folic acid supplementation on overall and site-specific cancer incidence during the randomised trials: meta-analyses of data on 50,000 individuals. Lancet.

[101] Qin X (2013). Folic acid supplementation and cancer risk: a meta-analysis of randomized controlled trials. Int J Cancer.

[102] Evans JR, Lawrenson JG (2017). Antioxidant vitamin and mineral supplements for preventing age-related macular degeneration. Cochrane Database Syst Rev.

[103] Patel KR, Sobczyńska-Malefora A (2017). The adverse effects of an excessive folic acid intake. Eur J Clin Nutr.

[104] Schmidl D (2020). A pilot study to assess the effect of a three-month vitamin supplementation containing L-methylfolate on systemic homocysteine plasma concentrations and retinal blood flow in patients with diabetes. Mol Vis.

[105] Title LM (2006). Folic acid improves endothelial dysfunction in type 2 diabetes–an effect independent of homocysteine-lowering. Vasc Med.

[106] Zamani M (2023). The effects of folic acid supplementation on endothelial function in adults: a systematic review and dose-response meta-analysis of randomized controlled trials. Nutr J.

